# Research Ethics Boards: Reply from Ezekiel Emanuel

**DOI:** 10.1371/journal.pmed.0030460

**Published:** 2006-10-31

**Authors:** Ezekiel J Emanuel

I have misstated. Obviously, as a governmental agency, the Office for Human Research Protections (OHRP) could not “endorse” the Western Institutional Review Board (WIRB) to re-review protocols at institutions whose federal assurance was suspended, or advise such institutions to consult WIRB. As Dr. Schwetz reminded me [1], OHRP could provide information about independent or other IRBs that could help suspended institutions re-review protocols, but could not suggest which one they should employ or endorse a particular IRB. I presume OHRP would not provide information to institutions on IRBs that it deemed to have questionable practices or performance in reviewing protocols. So while as a government agency it could not provide a formal endorsement, there is an implied claim that the IRBs mentioned by OHRP conduct satisfactory reviews. Furthermore, as a matter of fact it is worthy of note that the University of Rochester, the University of Colorado, Johns Hopkins University, and other academic institutions whose federal assurance was suspended by OHRP ended up consulting WIRB. I stand corrected.
